# 1-(2-Fluoro­benz­yl)-1-(2-fluoro­benz­yl­oxy)urea

**DOI:** 10.1107/S1600536809000622

**Published:** 2009-01-31

**Authors:** Xi Mai, Hong-Ying Xia, Yu-Sheng Cao, Xiao-San Lu, Xiao-Niu Fang

**Affiliations:** a Sino-German Joint Research Institute of Nanchang University, Nanchang 330006, People’s Republic of China; b Department of Pharmacy, Medical College of Nanchang University, Nanchang 330006, People’s Republic of China; c Department of Chemistry, Jinggangshan University, Ji’an 343009, People’s Republic of China

## Abstract

In the title hydroxy­urea derivative, C_15_H_14_F_2_N_2_O_2_, the dihedral angle between the two benzene rings is 48.64 (19)°. The urea group forms dihedral angles of 48.1 (2) and 79.2 (2)° with the two benzene rings. In the crystal, inversion dimers linked by pairs of N—H⋯O hydrogen bonds occur, and further N—H⋯O links lead to chains of molecules.

## Related literature

For geneal background, see: Krakoff *et al.* (1968 ▶); Young *et al.* (1967 ▶) and Yu *et al.* (1974 ▶). For related structures, see: Howard *et al.* (1967 ▶); Thiessen *et al.* (1978 ▶); Armagan *et al.* (1976 ▶); Berman & Kim (1967 ▶); Larsen *et al.* (1966 ▶); Nielsen *et al.* (1993 ▶).
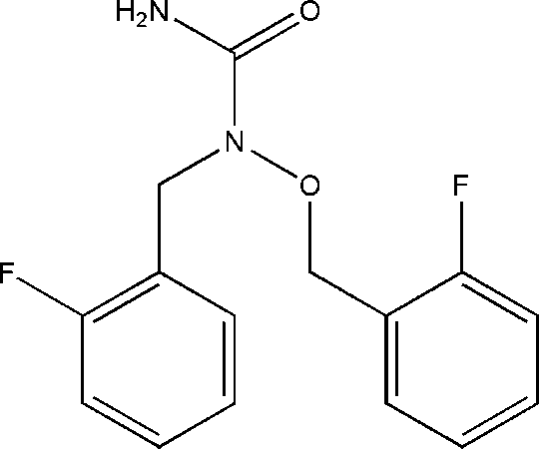


## Experimental

### 

#### Crystal data

C_15_H_14_F_2_N_2_O_2_
*M* *_r_* = 292.28
Monoclinic, 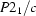

*a* = 5.196 (5) Å
*b* = 30.11 (3) Å
*c* = 9.059 (8) Å
β = 102.110 (16)°
*V* = 1386 (2) Å^3^
*Z* = 4
Mo *K*α radiation
μ = 0.11 mm^−1^
*T* = 296 (2) K
0.34 × 0.13 × 0.07 mm

#### Data collection

Bruker APEXII area-detector diffractometer
Absorption correction: none
8214 measured reflections
2416 independent reflections
1042 reflections with *I* > 2σ(*I*)
*R*_int_ = 0.051

#### Refinement

*R*[*F* ^2^ > 2σ(*F* ^2^)] = 0.080
*wR*(*F* ^2^) = 0.260
*S* = 1.02
2416 reflections
166 parameters
H-atom parameters constrained
Δρ_max_ = 0.39 e Å^−3^
Δρ_min_ = −0.37 e Å^−3^

### 

Data collection: *APEX2* (Bruker, 2004 ▶); cell refinement: *SAINT* (Bruker, 2004 ▶); data reduction: *SAINT*; program(s) used to solve structure: *SHELXS97* (Sheldrick, 2008 ▶); program(s) used to refine structure: *SHELXL97* (Sheldrick, 2008 ▶); molecular graphics: *ORTEP-3 for Windows* (Farrugia, 1997 ▶); software used to prepare material for publication: *publCIF* (Westrip, 2009 ▶).

## Supplementary Material

Crystal structure: contains datablocks I, global. DOI: 10.1107/S1600536809000622/xu2472sup1.cif

Structure factors: contains datablocks I. DOI: 10.1107/S1600536809000622/xu2472Isup2.hkl

Additional supplementary materials:  crystallographic information; 3D view; checkCIF report

## Figures and Tables

**Table 1:** Hydrogen-bond geometry (Å, °)

*D*—H⋯*A*	*D*—H	H⋯*A*	*D*⋯*A*	*D*—H⋯*A*
N2—H2*C*⋯O2^i^	0.86	2.05	2.910 (5)	174
N2—H2*D*⋯O2^ii^	0.86	2.32	3.079 (5)	148

Symmetry codes: (i) 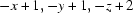; (ii) 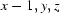.

**Fig. 1.**
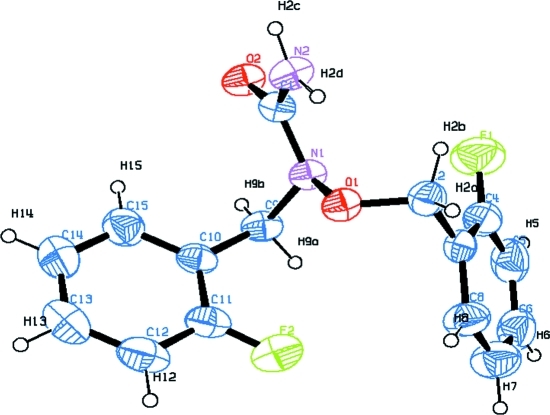
Molecular structure of the title compound showing the atom labeling scheme. Displacement ellipsoids are drawn at the 50% probability level.

## supplementary crystallographic information

### Comment

The anticancer drug hydroxyurea, which has been used in cancer chemotherapy for many years, has shown to impair DNA synthesis by inhibiting the enzyme ribonucleotide reductase (RNR) (Krakoff et al., 1968). Many hydroxyurea derivates has been designed and synthesized, which inhibit RNR by the same mechanism. We designed and synthesized N'-unsubstituted N-hydroxyure derivative, 1-(2-fluorobenzyl)-1-(2-fluorobenzyloxy)urea. Then we used the compound to make the antitumor activity test in vitro for lymphoid leukemia L1210 through the classic MTT assay. Results show that it has higher inhibition ratios than N-hydroxyurea. This seems to be not much in good agreement with the early structure-activity studies of Young et al. (1967) and Yu et al. (1974). As a serial study of such a complex, the title compound was synthesized and its crystal structure is reported here.

The conformations of the N—H and C=O bonds in the structure of 1-(2-fluorobenzyl)-1-(2-fluorobenzyloxy)urea (Fig. 1) are anti to each other, similar to that observed in N-hydroxyurea (Howard et al., 1967; Thiessen et al., 1978; Armagan et al., 1976; Berman & Kim, 1967; Larsen et al., 1966), 1-hydroxy-1-methylurea (Nielsen et al., 1993), 1-hydroxy-3-methylurea (Nielsen et al., 1993) and other hydroxyurea derivates. The bond parameters in N-(phenylmethoxy)-urea are similar to those in above hydroxyurea derivates, but the length of the carbonyl bond (C=O) is obviously shorter (< 1.25 Å). This may be related with the hydroxy group's etherification. The urea N—(C=O) —N group forms a dihedral angle of 48.1 (2) and 79.2 (2)° with the two benzene rings respectively. Intermolecular N—H···O hydrogen bonding presents in the crystal structure (Table 1).

### Experimental

The title compound was prepared by the reaction of 1-(2-fluorobenzyloxy)urea (1.3 mmol) and 1-(chloromethyl)-2-fluorobenzene (1.3 mmol) in methanol (10 ml) in the presence of potassium hydroxide (1.7 mmol). After refluxing for 14 h, the mixture was distilled in the reduced pressure at 308 K. The resulting crude solid was filtered and washed by trichloromethane repeatedly, then recrystallized in acetone and trichloromethane mixture (5:2), filtered. Colorless needle-shaped single crystals used for X-ray structure determination were recrystallized from the mixed solvent acetone and *N*-hexane (3:13) at room temperature for one week.

### Refinement

H atoms were placed in calculated positions with C—H = 0.93 (aromatic), 0.97 Å (methylene) and N—H = 0.86 Å, and were refined in riding mode. The U_iso_(H) values were set at 1.2U_eq_(C,N).

### Crystal data

**Table:**

C_15_H_14_F_2_N_2_O_2_	*F*(000) = 608
*M**_r_* = 292.28	*D*_x_ = 1.401 Mg m^−^^3^
Monoclinic, *P*2_1_/*c*	Melting point: 414.0 K
Hall symbol: -P 2ybc	Mo *K*α radiation, λ = 0.71073 Å
*a* = 5.196 (5) Å	Cell parameters from 1260 reflections
*b* = 30.11 (3) Å	θ = 2.4–19.3°
*c* = 9.059 (8) Å	µ = 0.11 mm^−^^1^
β = 102.110 (16)°	*T* = 296 K
*V* = 1386 (2) Å^3^	Needle, colourless
*Z* = 4	0.34 × 0.13 × 0.07 mm

### Data collection

**Table:**

Bruker APEXII area-detector diffractometer	1042 reflections with *I* > 2σ(*I*)
Radiation source: fine-focus sealed tube	*R*_int_ = 0.051
graphite	θ_max_ = 25.0°, θ_min_ = 2.4°
φ and ω scans	*h* = −6→6
8214 measured reflections	*k* = −35→35
2416 independent reflections	*l* = −10→10

### Refinement

**Table:**

Refinement on *F*^2^	Primary atom site location: structure-invariant direct methods
Least-squares matrix: full	Secondary atom site location: difference Fourier map
*R*[*F*^2^ > 2σ(*F*^2^)] = 0.080	Hydrogen site location: inferred from neighbouring sites
*wR*(*F*^2^) = 0.260	H-atom parameters constrained
*S* = 1.02	*w* = 1/[σ^2^(*F*_o_^2^) + (0.14*P*)^2^] where *P* = (*F*_o_^2^ + 2*F*_c_^2^)/3
2416 reflections	(Δ/σ)_max_ < 0.001
166 parameters	Δρ_max_ = 0.39 e Å^−^^3^
0 restraints	Δρ_min_ = −0.37 e Å^−^^3^

### Special details

**Table:**

Geometry. All e.s.d.'s (except the e.s.d. in the dihedral angle between two l.s. planes) are estimated using the full covariance matrix. The cell e.s.d.'s are taken into account individually in the estimation of e.s.d.'s in distances, angles and torsion angles; correlations between e.s.d.'s in cell parameters are only used when they are defined by crystal symmetry. An approximate (isotropic) treatment of cell e.s.d.'s is used for estimating e.s.d.'s involving l.s. planes.
Refinement. Refinement of *F*^2^ against ALL reflections. The weighted *R*-factor *wR* and goodness of fit *S* are based on *F*^2^, conventional *R*-factors *R* are based on *F*, with *F* set to zero for negative *F*^2^. The threshold expression of *F*^2^ > σ(*F*^2^) is used only for calculating *R*-factors(gt) *etc*. and is not relevant to the choice of reflections for refinement. *R*-factors based on *F*^2^ are statistically about twice as large as those based on *F*, and *R*- factors based on ALL data will be even larger.

### Fractional atomic coordinates and isotropic or equivalent isotropic displacement parameters (Å^2^)

**Table:**

	*x*	*y*	*z*	*U*_iso_*/*U*_eq_	
C10	0.2248 (6)	0.40316 (12)	0.5643 (3)	0.0675 (13)	
C11	−0.0040 (7)	0.38153 (11)	0.4930 (4)	0.0829 (15)	
C12	−0.1631 (6)	0.40007 (16)	0.3653 (4)	0.106 (2)	
H12	−0.3162	0.3856	0.3175	0.127*	
C13	−0.0935 (9)	0.44024 (17)	0.3089 (4)	0.118 (2)	
H13	−0.2000	0.4526	0.2235	0.141*	
C14	0.1352 (10)	0.46187 (12)	0.3803 (5)	0.119 (2)	
H14	0.1818	0.4887	0.3426	0.142*	
C15	0.2944 (7)	0.44333 (12)	0.5080 (5)	0.0938 (17)	
H15	0.4474	0.4578	0.5557	0.113*	
C3	0.1504 (7)	0.31233 (9)	0.9537 (4)	0.0677 (13)	
C4	0.3952 (7)	0.29896 (13)	1.0354 (4)	0.0867 (16)	
C5	0.4847 (6)	0.25614 (15)	1.0185 (5)	0.108 (2)	
H5	0.6484	0.2472	1.0732	0.130*	
C6	0.3294 (9)	0.22670 (10)	0.9199 (5)	0.1050 (19)	
H6	0.3893	0.1980	0.9086	0.126*	
C7	0.0846 (9)	0.24007 (11)	0.8383 (5)	0.113 (2)	
H7	−0.0192	0.2204	0.7723	0.135*	
C8	−0.0049 (6)	0.28289 (12)	0.8552 (4)	0.0964 (18)	
H8	−0.1686	0.2918	0.8005	0.116*	
C1	0.3969 (8)	0.43961 (14)	0.9001 (5)	0.0581 (11)	
C2	0.0492 (10)	0.35829 (16)	0.9702 (6)	0.0767 (14)	
H2A	−0.1322	0.3564	0.9812	0.092*	
H2B	0.1512	0.3716	1.0615	0.092*	
C9	0.3958 (9)	0.38360 (15)	0.7028 (5)	0.0675 (13)	
H9A	0.3850	0.3515	0.6953	0.081*	
H9B	0.5769	0.3919	0.7050	0.081*	
F1	0.5413 (8)	0.32477 (14)	1.1264 (5)	0.1447 (16)	
F2	−0.0789 (8)	0.34425 (11)	0.5430 (4)	0.1243 (13)	
N1	0.3293 (6)	0.39708 (11)	0.8446 (4)	0.0594 (10)	
N2	0.2206 (7)	0.46056 (11)	0.9610 (4)	0.0693 (11)	
H2C	0.2563	0.4863	1.0015	0.083*	
H2D	0.0704	0.4485	0.9601	0.083*	
O1	0.0624 (5)	0.38636 (9)	0.8448 (3)	0.0635 (9)	
O2	0.6157 (6)	0.45452 (10)	0.8966 (4)	0.0719 (10)	

### Atomic displacement parameters (Å^2^)

**Table:**

	*U*^11^	*U*^22^	*U*^33^	*U*^12^	*U*^13^	*U*^23^
C10	0.062 (3)	0.080 (3)	0.062 (3)	0.000 (3)	0.018 (2)	−0.019 (2)
C11	0.080 (4)	0.097 (4)	0.071 (4)	−0.010 (3)	0.014 (3)	−0.025 (3)
C12	0.095 (4)	0.139 (6)	0.080 (4)	−0.020 (4)	0.008 (4)	−0.029 (4)
C13	0.132 (6)	0.145 (6)	0.070 (4)	0.005 (5)	0.006 (4)	0.003 (4)
C14	0.141 (6)	0.123 (5)	0.084 (5)	−0.021 (5)	0.006 (4)	0.016 (4)
C15	0.092 (4)	0.111 (4)	0.080 (4)	−0.015 (3)	0.020 (3)	−0.001 (3)
C3	0.071 (3)	0.072 (3)	0.064 (3)	−0.015 (3)	0.021 (3)	0.002 (2)
C4	0.084 (4)	0.091 (4)	0.077 (4)	−0.011 (3)	−0.003 (3)	0.006 (3)
C5	0.106 (5)	0.111 (5)	0.102 (5)	0.010 (4)	0.007 (4)	0.036 (4)
C6	0.127 (5)	0.083 (4)	0.108 (5)	0.007 (4)	0.031 (4)	0.016 (4)
C7	0.106 (5)	0.097 (5)	0.121 (5)	0.000 (4)	−0.009 (4)	−0.008 (4)
C8	0.108 (4)	0.067 (4)	0.104 (4)	−0.001 (3)	0.000 (3)	−0.011 (3)
C1	0.050 (3)	0.064 (3)	0.059 (3)	0.004 (2)	0.008 (2)	−0.006 (2)
C2	0.075 (3)	0.082 (3)	0.076 (3)	−0.015 (3)	0.023 (3)	−0.006 (3)
C9	0.056 (3)	0.070 (3)	0.080 (3)	0.004 (2)	0.022 (2)	−0.021 (2)
F1	0.140 (3)	0.135 (3)	0.133 (3)	−0.026 (3)	−0.031 (2)	−0.013 (2)
F2	0.134 (3)	0.105 (3)	0.129 (3)	−0.038 (2)	0.016 (2)	−0.019 (2)
N1	0.049 (2)	0.061 (2)	0.069 (2)	−0.0016 (16)	0.0145 (17)	−0.0087 (18)
N2	0.054 (2)	0.063 (2)	0.096 (3)	−0.0041 (18)	0.027 (2)	−0.018 (2)
O1	0.0480 (17)	0.0676 (19)	0.077 (2)	−0.0041 (14)	0.0176 (15)	−0.0076 (15)
O2	0.0490 (18)	0.074 (2)	0.095 (2)	−0.0078 (16)	0.0216 (16)	−0.0186 (16)

### Geometric parameters (Å, °)

**Table:**

C10—C11	1.3900	C5—H5	0.9300
C10—C15	1.3900	C6—C7	1.3900
C10—C9	1.497 (6)	C6—H6	0.9300
C11—F2	1.301 (4)	C7—C8	1.3900
C11—C12	1.3900	C7—H7	0.9300
C12—C13	1.3900	C8—H8	0.9300
C12—H12	0.9300	C1—O2	1.229 (5)
C13—C14	1.3900	C1—N2	1.324 (5)
C13—H13	0.9300	C1—N1	1.394 (5)
C14—C15	1.3900	C2—O1	1.429 (6)
C14—H14	0.9300	C2—H2A	0.9700
C15—H15	0.9300	C2—H2B	0.9700
C3—C4	1.3900	C9—N1	1.457 (6)
C3—C8	1.3900	C9—H9A	0.9700
C3—C2	1.499 (6)	C9—H9B	0.9700
C4—F1	1.264 (4)	N1—O1	1.424 (4)
C4—C5	1.3900	N2—H2C	0.8600
C5—C6	1.3900	N2—H2D	0.8600
			
C11—C10—C15	120.0	C5—C6—H6	120.0
C11—C10—C9	120.3 (3)	C6—C7—C8	120.0
C15—C10—C9	119.7 (3)	C6—C7—H7	120.0
F2—C11—C12	117.9 (3)	C8—C7—H7	120.0
F2—C11—C10	122.1 (3)	C7—C8—C3	120.0
C12—C11—C10	120.0	C7—C8—H8	120.0
C11—C12—C13	120.0	C3—C8—H8	120.0
C11—C12—H12	120.0	O2—C1—N2	124.2 (4)
C13—C12—H12	120.0	O2—C1—N1	119.4 (4)
C14—C13—C12	120.0	N2—C1—N1	116.4 (4)
C14—C13—H13	120.0	O1—C2—C3	113.0 (4)
C12—C13—H13	120.0	O1—C2—H2A	109.0
C13—C14—C15	120.0	C3—C2—H2A	109.0
C13—C14—H14	120.0	O1—C2—H2B	109.0
C15—C14—H14	120.0	C3—C2—H2B	109.0
C14—C15—C10	120.0	H2A—C2—H2B	107.8
C14—C15—H15	120.0	N1—C9—C10	114.9 (3)
C10—C15—H15	120.0	N1—C9—H9A	108.6
C4—C3—C8	120.0	C10—C9—H9A	108.6
C4—C3—C2	121.0 (3)	N1—C9—H9B	108.6
C8—C3—C2	119.0 (3)	C10—C9—H9B	108.6
F1—C4—C5	118.2 (4)	H9A—C9—H9B	107.5
F1—C4—C3	121.8 (4)	C1—N1—O1	112.3 (3)
C5—C4—C3	120.0	C1—N1—C9	119.0 (4)
C4—C5—C6	120.0	O1—N1—C9	110.4 (3)
C4—C5—H5	120.0	C1—N2—H2C	120.0
C6—C5—H5	120.0	C1—N2—H2D	120.0
C7—C6—C5	120.0	H2C—N2—H2D	120.0
C7—C6—H6	120.0	N1—O1—C2	110.1 (3)

### Hydrogen-bond geometry (Å, °)

**Table:**

*D*—H···*A*	*D*—H	H···*A*	*D*···*A*	*D*—H···*A*
N2—H2C···O2^i^	0.86	2.05	2.910 (5)	174
N2—H2D···O2^ii^	0.86	2.32	3.079 (5)	148

Symmetry codes: (i) −*x*+1, −*y*+1, −*z*+2; (ii) *x*−1, *y*, *z*.
